# Proteomics uncovers molecular features for relapse risk stratification in patients with diffuse large B-cell lymphoma

**DOI:** 10.1038/s41408-023-00931-6

**Published:** 2023-10-26

**Authors:** Maja Ludvigsen, Amanda Jessica Campbell, Marie Beck Enemark, Trine Engelbrecht Hybel, Marja-Liisa Karjalainen-Lindsberg, Klaus Beiske, Mette Bjerre, Lars Møller Pedersen, Harald Holte, Sirpa Leppä, Judit Meszaros Jørgensen, Bent Honoré

**Affiliations:** 1 Department of Hematology, Aarhus University Hospital, Aarhus, Denmark; 2 Department of Clinical Medicine, Aarhus University, Aarhus, Denmark; 3 Department of Pathology, Helsinki University Hospital, Helsinki, Finland; 4 Department of Pathology, Division of Cancer Medicine, Oslo University Hospital, Oslo, Norway; 5 Medical/Steno Aarhus Research Laboratory, Department of Clinical Medicine, Aarhus University, Aarhus, Denmark; 6 Zealand University Hospital, Roskilde, Denmark; 7 Department of Oncology, Oslo University Hospital, Oslo, Norway; 8 KG Jebsen Center for B-Cell Malignancies, Institute of Clinical Medicine, University of Oslo, Oslo, Norway; 9 Norwegian Cancer Genomics Consortium, Oslo, Norway; 10 Research Program Unit, Applied Tumor Genomics, Faculty of Medicine, University of Helsinki, Helsinki, Finland; 11 Department of Oncology, Helsinki University Hospital Comprehensive Cancer Center, Helsinki, Finland; 12 iCAN Digital Precision Cancer Medicine Flagship, Helsinki, Finland; 13 Department of Biomedicine, Aarhus University, Aarhus, Denmark

**Keywords:** Hodgkin lymphoma, Translational research

Dear Editor,

Diffuse large B-cell lymphoma (DLBCL) is the most common lymphoid cancer, constituting approximately one third of all cases [1]. Although immunochemotherapy cures ~60–75% of patients, a significant proportion of patients still experience relapse [2, 3]. Relapsed DLBCL patients have poor outcomes, with a median overall survival of only 6.3 months with conventional chemotherapy, exemplifying the need for improved treatment options for these patients [4]. The ability to identify these patients at diagnosis using molecular markers is imperative and presently lacking in the clinic. Currently, cell-of-origin (COO) subtyping is widely used in clinical practice, which separates tumors with activated B cell-like (ABC) and germinal center B cell-like (GCB) gene expression signatures, with the ABC subtype having significantly worse survival [5, 6]. Additionally, double or triple-hit lymphomas, characterized by *MYC* and *BCL2* and/or *BCL6* translocations, *MYC* rearrangements, and *TP53* mutations are associated with poorer prognosis [7, 8]. Although these may help to identify patients with adverse prognosis, no biological markers have yet been identified to specifically identify patients at higher risk of relapse. Currently, the proteome of DLBCL is less well-studied. Various factors may cause discrepancies between gene expression and the final protein product. Therefore, a better understanding of the proteome of DLBCL could provide further insight into the biological mechanisms that drive relapse of DLBCL.

In this study, we used high-throughput mass spectrometry (MS) based proteomics to identify differentially expressed proteins in pretreatment lymphoma-tissue and serum samples. To identify potential biomarker candidates, we compared the protein profiles between subsequently relapsing (further denoted “relapsing”) and treatment-sensitive DLBCLs. Included patients were treated in two phase II clinical trials conducted by the Nordic Lymphoma Group (NCT01502982/NCT01325194) [9, 10]. Diagnostic formalin-fixed paraffin-embedded (FFPE) lymphoma tissue from patients were available, of which a subset had also a pretreatment serum sample available. A detailed description of inclusion criteria is specified in Supplementary Methods. While all patients initially responded, in the relapsing group patients experienced relapse within two years of follow-up. Analyses of lymphoma samples included 53 treatment-sensitive and 11 relapsing samples (Table S1A). Analyses of serum samples included 24 treatment-sensitive and 7 relapsing samples (Table S1B). To identify differentially expressed proteins, a label-free quantification nano liquid chromatography-tandem MS (LFQ nLC-MS/MS)-based proteomic analysis was performed. All procedures are described in detail in the Supplementary Methods. In addition, serum samples were included and enriched for low-abundance proteins. Bioinformatic analyses were performed using the Ingenuity Pathway Analysis (IPA) (QIAGEN Inc., Hilden, Germany). Selected proteins identified by proteomics were evaluated using enzyme-linked immunosorbent assay (ELISA) commercial kits.

Protein expression in both lymphoma and serum samples was compared between relapsing and treatment-sensitive patients (Fig. S1). Overall, clinicopathological features between the groups were similar (Tables S1A, B). In lymphoma tissues, 2026 proteins were detected in at least 70% of samples in each group. From these, 190 were significantly differentially expressed between relapsing and treatment-sensitive samples (Table S2), 149 being upregulated (fold changes 1.22–2.40) in relapsing samples, including TP53RK, RAC1/3, ARHGAP17, CDK2, as well as several ribosomal proteins, and 41 being downregulated (fold changes 0.28–0.79) including hexokinase 3, LDH a-chain, and GAPDH (Fig. 1A). Unsupervised principal component analysis (PCA) using the significantly differentially expressed proteins revealed a strong focusing of most relapsing samples with treatment-sensitive samples intermingled (Fig. 1B). Hierarchical clustering showed that all but one relapsing sample were in a cluster of 19 patients; 10 relapsing and 9 sensitive patients, and this was designated as a high-risk group (Fig. 1C). This high-risk group was reanalyzed separately, and here 2123 proteins were detected with 64 proteins significantly differentially expressed, 43 upregulated (fold changes 1.25–3.84) and 21 downregulated (fold changes 0.37–0.79) in the relapsing tumors (Fig. 1D, Table S3). PCA and hierarchical clustering using significantly differentially expressed proteins were able to fully discriminate between the relapsing and treatment-sensitive lymphomas (Fig. 1E, F).

**Fig. 1:**
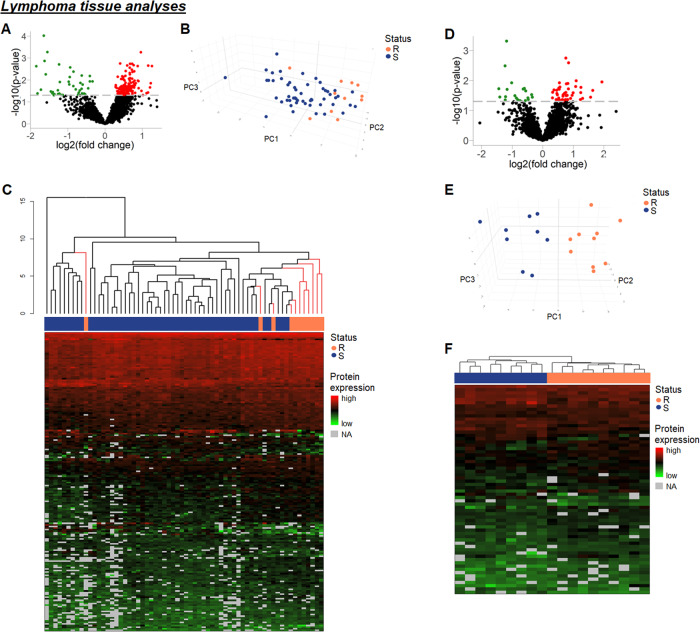
Analyses of lymphoma tissue samples. **A** Volcano plot showing 2026 proteins identified in at least 70% of lymphoma samples in each response group. 190 of these proteins were differentially expressed between relapsing and treatment-sensitive lymphomas. Red, upregulated; green, downregulated (*p* < 0.05). **B** 3D PCA of 82 differentially expressed proteins that were present in all samples. **C** Dendrogram showing hierarchical clustering of lymphomas based on 190 differentially expressed proteins. Black lines, treatment-sensitive lymphomas; pink lines, relapsing lymphomas. **D** Analysis of lymphoma tissue from high-risk cluster. Volcano plot showing 2123 proteins identified in at least 70% of lymphoma samples in each response group. 64 of these proteins were differentially expressed between relapsing and treatment-sensitive tumors. Red, upregulated; green, downregulated (*p* < 0.05). **E** 3D PCA of 37 differentially expressed proteins that were present in all samples, showing separation of relapsing and treatment-sensitive lymphomas. **F** Heatmap visualizing differentially expressed proteins between relapsing and treatment-sensitive lymphoma tissue in the high-risk cluster. 64 differentially expressed proteins in lymphoma tissue in the high-risk cluster (*n* = 19), separating relapsing samples (*n* = 10) from treatment-sensitive samples (*n* = 9). Each row is a protein and each column is a lymphoma sample. Above each heatmap is a dendrogram produced by hierarchical clustering using the proteins shown. A full list of the proteins can be found in the Supplementary Tables. Black lines, treatment-sensitive lymphomas; pink lines, relapsing lymphomas; NA not applicable (indicates proteins with missing values). R relapsing lymphomas; S treatment-sensitive lymphomas.

Pathway analysis of 190 significantly differentially expressed proteins revealed disturbances in important cellular pathways including protein synthesis (EIF2 signaling) and tumor suppression (Table S4). An IPA disease and function as well as network analysis revealed significant inhibition of tumor cell death (z-score ≤ −2) and disturbances in cell cycle regulation, RNA metabolism, and protein synthesis (Tables S5, S6), suggesting altered cell survival. Analysis of the high-risk subgroup further revealed significant stimulation of xenobiotic drug metabolism in relapsing tumors (z-score ≥ 2), indicating an increased metabolic rate of cytotoxic drugs in relapsing patients (Table S7). Furthermore, the IPA analyses revealed dysregulated cell death/survival signaling, as well as malignant invasion pathways (Tables S8, S9).

Analysis of serum samples identified 298 proteins in at least 70% of samples in each group. Of these, 20 proteins were significantly differentially expressed (Table S10), 15 of which were upregulated (fold changes 1.60–5.72), including DKK3, FCN3, and SAA1, while 5 were downregulated (fold changes 0.18–0.57), including lactotransferrin and angiogenin, in relapsing samples (Fig. 2A). PCA and hierarchal clustering with input of differentially expressed proteins revealed clear separation of relapsing and treatment-sensitive cases (Fig. 2B, C). Despite significant differences in protein expression, the 20 differentially expressed proteins also revealed high deviation in expression levels within the groups (Fig. 2D), highlighting the high heterogeneity in the disease across samples. Pathway analysis of the 20 differentially expressed proteins revealed cytokine-mediated disturbance of the negative hepatic acute phase response and dysregulation of cell senescence pathways (Table S11). IPA network analysis showed dysregulation of lipid metabolism as well as cell death/survival (Table S12). Additionally, LPS-IL-1-mediated inhibition of RXR function was also perturbed. Although based on proteins different to those in the tumor-tissue analysis, this pathway also relates to xenobiotic drug metabolism, supporting the hypothesis of an altered cytotoxic drug metabolism. Selected proteins, DKK3, FCN3, and SAA, were further evaluated using ELISA. In all cases, median protein expression was higher in the relapsing group than in the treatment-sensitive group, however, without reaching statistical significance (Fig. S2B).

**Fig. 2:**
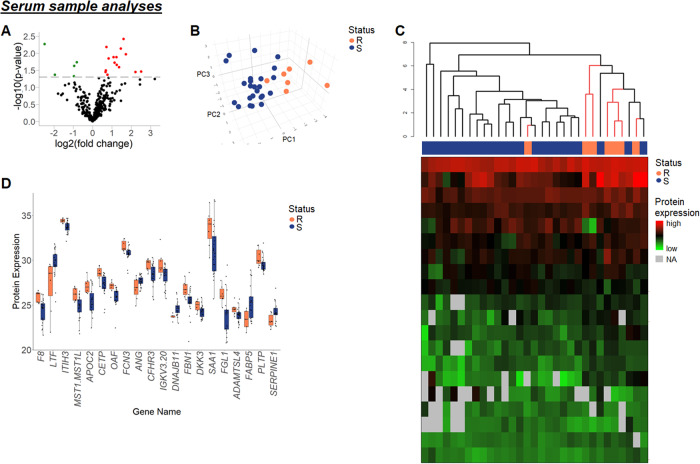
Analysis of serum samples. **A** Volcano plot showing 298 proteins identified in at least 70% of serum samples in each response group. 20 of these proteins were differentially expressed between relapsing and treatment-sensitive samples. Red, upregulated; green, downregulated (*p* < 0.05). **B** 3D PCA of 12 differentially expressed serum proteins that were present in all samples. **C** Dendrogram showing hierarchical clustering of samples based on 20 differentially expressed serum proteins. Black lines, treatment-sensitive lymphomas; pink lines, relapsing lymphomas. **D** Boxplot of 20 differentially expressed proteins in serum samples from relapsing and treatment-sensitive patients. Proteins are referred to by their gene names for simplicity. R relapsing lymphomas; S treatment-sensitive lymphomas.

Taken together, analyzing diagnostic FFPE lymphoma samples and protein-enriched serum samples from DLBCL patients revealed different protein expression profiles according to clinical outcome. Currently, clinical outcomes vary widely among DLBCL patients, and the ability to accurately identify patients with high risk of relapse remains an unmet clinical need. Using high-throughput proteomics, this study has identified protein candidates that can be further investigated as putative prognostic biomarkers. While differentially expressed proteins in the lymphomas showed strong focus of relapsing patients in clustering analyses, differentially expressed proteins from serum samples enabled almost distinct clustering of samples corresponding to relapsing and treatment-sensitive lymphomas.

Upregulated proteins included the TP53-regulating TP53RK and several members of the Rho family of GTPases, which are important for the invasion abilities of malignant cells [11]. Going forward, these highlighted proteins should be validated in larger and independent cohorts to explore clinical applications. In previous studies, proteomic investigation in the context of relapsed DLBCL patients revealed dysregulation of ribosomal, inflammation and immune-related protein networks [12–14]. In the present study, of 149 significantly upregulated proteins, 44 were ribosomal proteins. It is unclear whether the upregulation of ribosomal proteins observed in relapsing DLBCL is due to increased translation activity or the ribosome-independent functions of ribosomal proteins, and thus, functional studies are needed to clarify this point.

Importantly, this study has shown that protein expression in diagnostic serum samples differs between relapsing and treatment-sensitive patients. Among differentially expressed proteins were SAA, isoform 1. SAA is upregulated during the acute phase response to inflammation and has also been found elevated in many pathological conditions, including cancer [15]. SAA was one of the proteins selected for validation by an ELISA analysis, however, the commercial kit did not distinguish between different isoforms of SAA. Pathway analyses of both tumor tissue samples and serum samples showed perturbances regarding xenobiotic metabolism, hereby suggesting an altered cytotoxic drug metabolism, which may lead to inferior performance in this group. Interestingly, this was the only pathway identified in both lymphoma-tissue and serum-sample analyses. This could be due to technical reasons. Alternatively, the protein changes observed in the tumor-tissue biopsies could mirror the underlying tumor biology to a larger extend than changes observed in the blood due to systemic “dilution”. However, further studies are warranted to further characterize the lymphoma-tissue/serum proteome of DLBCL.

MS-based proteomics results in serum samples were partially validated by ELISA, where similar trends in protein expression levels were observed, although not statistically significant, possibly because the MS analyses were based on serum samples being pre-treated to enable enrichment of low-abundant proteins whereas the ELISA was performed directly on the serum samples. This was done with the perspective to avoid the enrichment step if such an analysis should be used in a future diagnostic setting. In this exploratory study, p-values were not corrected for multiple testing. Although this may increase the number of false positives, false negatives are kept at a lower number, and therefore, potentially important proteins are less likely to be missed.

In conclusion, the purpose of this exploratory study was to characterize the proteome of DLBCL according to risk of relapse and certainly, results from this study constitutes a steppingstone to develop further studies allowing more detailed characterization of pivotal proteins in the pathogenesis of DLBCL.

### Supplementary information

Supplement material

Supplementary Figure 1

Supplementary Figure 2

Supplement Tables S5, S6, S8, S9, S12

## Data Availability

Data included in the current study are available upon reasonable request.

## References

[1] Shankland KR, Armitage JO, Hancock BW (2012). Non-Hodgkin lymphoma. Lancet [Internet].

[2] Pfreundschuh M, Schubert J, Ziepert M, Schmits R, Mohren M, Lengfelder E (2008). Six versus eight cycles of bi-weekly CHOP-14 with or without rituximab in elderly patients with aggressive CD20+ B-cell lymphomas: a randomised controlled trial (RICOVER-60). Lancet Oncol.

[3] Longo DL, Sehn LH, Salles G (2021). Diffuse Large B-Cell Lymphoma. N Engl J Med.

[4] Crump M, Neelapu SS, Farooq U, Van Den Neste E, Kuruvilla J, Westin J (2017). Outcomes in refractory diffuse large B-cell lymphoma: Results from the international SCHOLAR-1 study. Blood..

[5] Alizadeh AA, Elsen MB, Davis RE, Ma CL, Lossos IS, Rosenwald A (2000). Distinct types of diffuse large B-cell lymphoma identified by gene expression profiling. Nature.

[6] Scott DW, Wright GW, Williams PM, Lih CJ, Walsh W, Jaffe ES (2014). Determining cell-of-origin subtypes of diffuse large B-cell lymphoma using gene expression in formalin-fixed paraffin-embedded tissue. Blood.

[7] Barrans S, Crouch S, Smith A, Turner K, Owen R, Patmore R (2010). Rearrangement of MYC is associated with poor prognosis in patients with diffuse large B-cell lymphoma treated in the era of rituximab. J Clin Oncol.

[8] Deng M, Xu-Monette ZY, Pham LV, Wang X, Tzankov A, Fang X (2021). Aggressive B-cell lymphoma with MYC/TP53 dual alterations displays distinct clinicopathobiological features and response to novel targeted agents. Mol Cancer Res.

[9] Leppä S, Jørgensen J, Tierens A, Meriranta L, Østlie I, de Nully Brown P (2020). Patients with high-risk DLBCL benefit from dose-dense immunochemotherapy combined with early systemic CNS prophylaxis. Blood Adv.

[10] Holte H, Leppä S, Björkholm M, Fluge, Jyrkkiö S, Delabie J (2013). Dose-densified chemoimmunotherapy followed by systemic central nervous system prophylaxis for younger high-risk diffuse large B-cell/follicular grade 3 lymphoma patients: Results of a phase II Nordic lymphoma group study. Ann Oncol [Internet].

[11] Chan AY, Coniglio SJ, Chuang YY, Michaelson D, Knaus UG, Philips MR (2005). Roles of the Rac1 and Rac3 GTPases in human tumor cell invasion. Oncogene.

[12] Rüetschi U, Stenson M, Hasselblom S, Nilsson-Ehle H, Hansson U, Fagman H (2015). SILAC-based quantitative proteomic analysis of diffuse large B-cell lymphoma patients. Int J Proteom.

[13] Fornecker LM, Muller L, Bertrand F, Paul N, Pichot A, Herbrecht R (2019). Multi-omics dataset to decipher the complexity of drug resistance in diffuse large B-cell lymphoma. Sci Rep.

[14] Bram Ednersson S, Stenson M, Stern M, Enblad G, Fagman H, Nilsson-Ehle H (2018). Expression of ribosomal and actin network proteins and immunochemotherapy resistance in diffuse large B cell lymphoma patients. Br J Haematol.

[15] Buck M, Gouwy M, Wang J, Snick J, Opdenakker G, Struyf S (2016). Structure and expression of different serum amyloid A (SAA) variants and their concentration-dependent functions during host insults. Curr Med Chem.

