# Supplementary material for: Proteomics uncovers molecular features for relapse risk stratification in patients with diffuse large B-cell lymphoma
Source: Blood Cancer J. 2023 Oct 26;13(1):161. doi: 10.1038/s41408-023-00931-6 (PMC10603067; doi:10.1038/s41408-023-00931-6)
Supplement: Supplementary file 1 — Supplement material [file 41408_2023_931_MOESM1_ESM.docx]

**Supplementary Information: Proteomics uncovers molecular features for relapse risk stratification in patients with diffuse large B-cell lymphoma.**

**Supplementary Methods**

**Patients**

Patients were treated in two phase II clinical trials conducted by Nordic Lymphoma Group, NLG-LBC-04 (NCT01502982) and NLG-LBC-05 (NCT01325194) (1,2). Diagnostic formalin-fixed paraffin-embedded (FFPE) lymphoma tissue from 16 patients from the LBC-04 and 50 patients from the LBC-05 trials were available for the analysis. Of the 66 patients, 31 patients also had a pretreatment serum sample available. Inclusion criteria were age 18-64 years, histologically confirmed CD20+ DLBCL or FL grade 3B, World Health Organization performance status < 4, age adjusted IPI score 2-3, and no clinical, radiological, or cytological signs of central nervous system (CNS) involvement. Patients were treated with six cycles of R-CHOP-14 with etoposide along with CNS targeted therapy (high-dose methotrexate, high-dose cytarabine and in LBC-05 trial also intrathecally administered liposomal cytarabine) either at the end or in the beginning of immunochemotherapy. Radiotherapy was administered as needed. Patients were included from Finland and Norway. The three-year OS was 81% for LBC-04 and 86% for LBC-05, and, interestingly, DH/TH had no negative impact on prognosis in the LBC-05 study (1). To define response groups, relapsing patients were designated as patients, who initially responded (complete response, complete response unconfirmed, or partial response), but subsequently relapsed within two years of follow-up. Treatment-sensitive patients achieved complete response, complete response unconfirmed, or partial response and did not experience relapse within two years of follow-up. The present study was approved by the Regional Research Ethics Committee in Denmark (1-10-72-108-15) and the Danish Data Agency (1-16-02-267-15) and conducted in compliance with the principles of the Helsinki Declaration. The protocols were approved by the medical agencies and ethics committees in Finland, Denmark, Norway and Sweden and the trials were registered at ClinicalTrials.gov (Protocol Code NLG-LBC05, EUDRACT No 2010-023125-38; Protocol Code NLG-LBC-04, EUDRACT Number: 2004-003075-37). All patients signed informed consent before trial participation.

**FFPE tissue preparation**

Seven 10 µm sections of FFPE lymphoma tissue from each patient were deparaffinized and processed for proteomic analysis essentially as described (3), except that the rehydrated tissue was lysed in 1 x lysis buffer (5% sodium dodecyl sulphate [SDS], 50 mM triethylammonium bicarbonate [TEAB], pH 8.5). Protein concentration was measured by infrared spectrometry (27).

**Protein enrichment of serum samples for proteomic analysis**

Serum samples were enriched for low abundance proteins with the ProteoMiner^TM^ Sequential Elution Small-Capacity Protein Enrichment Kit (Bio-Rad Laboratories, Inc. Hercules, CA, USA) as described by the manufacturer. In short, for each sample 200 µL serum was added to the columns and four fractions were obtained by eluting firstly with 1 M sodium chloride, 20 mM HEPES, pH 7.4, secondly with 200 mM glycine, pH 2.4, thirdly with 60% ethylene glycol in water and finally with 33.3% 2-propanol, 16.7% acetonitrile, 0.1% trifluoroacetic acid. Each of the four fractions was precipitated by adding 5 volumes of ice-cold acetone at -20°C overnight and then dissolved in 1x lysis buffer. Protein concentration was measured by infrared spectrometry (4).

**Tryptic digestion of samples**

Up to 50 µg protein was tryptic digested with the suspension trapping method (5) using S-Trap micro columns from Protifi (Farmingdale, NY, USA) as previously described (6). The peptide concentration was measured by tryptophan fluorescence as described (4) and 1 µg of each sample was analyzed by nLC-MS/MS using a mass spectrometry platform consisting of an Ultimate 3000 nanoLC connected to an Orbitrap Fusion Tribrid mass spectrometer (Thermo Fisher Scientific, Waltham, MA, USA) with the settings for the universal method as previously described (6). FFPE analysis generated 124 raw files, 56 run as duplicates and 12 as single determinations, and serum analysis generated 244 raw files, 120 run as duplicates and 4 run as single determinations. The raw files were analyzed with MaxQuant version 1.6.6.0 (7). In the FFPE analysis the UniProt Homo sapiens database was downloaded 9 February 2020 and for the serum analysis the UniProt Homo sapiens database was downloaded 8 November 2020.

**Data and Bioinformatic Analyses**

Sixty-six FFPE samples and 31 protein enriched serum samples were analyzed in Perseus (version 1.6.15.0) (8). During preliminary principal components analysis (PCA) of FFPE tumor tissue, two outliers were identified (Figure S2A). In addition to their placement on the PCA plot, the outliers also had a high number of missing values. Therefore, due to their low data quality they were removed from the dataset, leaving 64 samples of tumor tissue available for analysis. P-values were calculated by two-tailed t-test without further correction in order not to increase type 2 errors with the risk of overlooking putative predictive markers. Data were analyzed with the use of QIAGEN IPA (QIAGEN Inc., <https://www.digitalinsights.qiagen.com/IPA>). The algorithms developed for use in QIAGEN IPA were described by Krämer et al. (9).

**Enzyme-linked Immunosorbent Assay**

Protein targets were selected for validation via enzyme-linked immunosorbent assay (ELISA) based on protein function, MS proteomics data and the availability of commercial sandwich ELISA kits. ELISA for Dickkopf-related protein 3 (DKK3) (RAB0145, Sigma-Aldrich, Saint Louis, MO, USA), ficolin-3 (FCN3) (HK340, Hycult Biotech Inc, Uden, Netherlands), and serum amyloid A (SAA) (RAB0420, Sigma-Aldrich, Saint Louis, MO, USA) were measured in serum samples according to the manufacturer.

**Statistical Analysis**

Differences in clinicopathological features between patients were assessed using χ^2-test and Fischer’s exact test. Student’s t-test was used to test for proteins differentially expressed in tumor tissue and serum samples from relapsing and treatment-sensitive patients. For PCA, all proteins containing missing values in some of the samples were removed from the data to avoid imputation and variables were scaled to a standard normal distribution. For hierarchical clustering, Euclidean distance was used as the dissimilarity measure and average linkage was used to join clusters. To evaluate whether proteins measured with ELISA were differentially expressed in relapsing and treatment-sensitive patients, Student’s t-test was used if the data passed the Shapiro-Wilk normality test and F-test for equal variance. Otherwise, nonparametric Mann Whitney U test was used.

P-values < 0.05 were considered statistically significant. Statistical analyses were performed in RStudio (R version 4.0.4, RStudio version 1.4.1106) (10,11).

References

1. Leppä S, Jørgensen J, Tierens A, Meriranta L, Østlie I, de Nully Brown P, et al. Patients with high-risk DLBCL benefit from dose-dense immunochemotherapy combined with early systemic CNS prophylaxis. Blood Adv. 2020;4(9):1906–15.
2. Holte H, Leppä S, Björkholm M, Fluge, Jyrkkiö S, Delabie J, et al. Dose-densified chemoimmunotherapy followed by systemic central nervous system prophylaxis for younger high-risk diffuse large B-cell/follicular grade 3 lymphoma patients: Results of a phase II Nordic lymphoma group study. Ann Oncol [Internet]. 2013;24(5):1385–92. Available from: <https://doi.org/10.1093/annonc/mds621>
3. Honoré B, Andersen MD, Wilken D, et al. Classic Hodgkin Lymphoma Refractory for ABVD Treatment Is Characterized by Pathologically Activated Signal Transduction Pathways as Revealed by Proteomic Profiling. Cancers (Basel). 2022;14(1).
4. Honoré B. Proteomic Protocols for Differential Protein Expression Analyses. In: Costa C, editor. Xenotransplantation: Methods and Protocols [Internet]. New York, NY: Springer US; 2020. p. 47–58. Available from: <https://doi.org/10.1007/978-1-0716-0255-3_3>
5. Zougman A, Selby PJ, Banks RE. Suspension trapping (STrap) sample preparation method for bottom-up proteomics analysis. Proteomics. 2014 May;14(9):1000–6.
6. Cehofski LJ, Kojima K, Terao N, Kitazawa K, Thineshkumar S, Grauslund J, et al. Aqueous Fibronectin Correlates With Severity of Macular Edema and Visual Acuity in Patients With Branch Retinal Vein Occlusion: A Proteome Study. Invest Ophthalmol Vis Sci [Internet]. 2020;61(14):6. Available from: <https://doi.org/10.1167/iovs.61.14.6>
7. Tyanova S, Temu T, Cox J. The MaxQuant computational platform for mass spectrometry-based shotgun proteomics. Nat Protoc [Internet]. 2016;11(12):2301–19. Available from: <https://doi.org/10.1038/nprot.2016.136>
8. Tyanova S, Temu T, Sinitcyn P, Carlson A, Hein MY, Geiger T, et al. The Perseus computational platform for comprehensive analysis of (prote)omics data. Nat Methods [Internet]. 2016;13(9):731–40. Available from: <https://doi.org/10.1038/nmeth.3901>
9. Krämer A, Green J, Pollard JJ, Tugendreich S. Causal analysis approaches in Ingenuity Pathway Analysis. Bioinformatics. 2014 Feb;30(4):523–30.
10. R Core Team. R: A language and environment for statistical computing [Internet]. R Foundation for Statistical Computing. 2021. Available from: <https://www.r-project.org/>
11. RStudio Team. RStudio: Integrated Development Environment for R [Internet]. RStudio, PBC. 2021. Available from: http://www.rstudio.com/

**Figure S1:** Flow diagram demonstrating workflow of the study. Workflow of study for lymphoma tissue (n=64) and protein-enriched serum samples (n=31). Pre-therapeutic samples were divided into subsequently relapsing and treatment-sensitive samples, respectively. Samples were then subjected to liquid chromatography and mass spectrometry-based proteomics for identification of differentially expressed proteins between the response groups. Subsequent statistical and bioinformatic analyses resulted in identification of risk groups of subsequent relapse. FFPE, formalin-fixed paraffin-embedded; LFQ nLC MS/MS, label-free quantification nano liquid chromatography-tandem mass spectrometry. Created in BioRender.com.

**Figure S2:** (A) 3D PCA plot of tumor tissue from all patients (n=66). All missing values were filtered from the data (664 proteins). Outliers are clearly visible on the far right and were removed from the dataset. R, relapse; S, treatment-sensitive. (B) Concentrations of DKK3, FCN3 and SAA in serum measured with ELISA. Although the median protein expression is higher in relapsing patients than treatment-sensitive patients for all protein targets, this trend did not reach statistical significance. For DKK3 and FCN3, nonparametric Mann Whitney U test was used to test for differential protein expression between relapsing and treatment-sensitive patients. For SAA, three samples were removed due to high variability between duplicates (CV>15%) and Student’s t-test was used. All removed samples were from the treatment-sensitive group. R, relapse; S, treatment-sensitive.

**Table S1: Clinicopathological features of patients that provided (A) tumor-tissue sample, and (B) a serum sample.**

| **A)** | **Characteristics** | **All, n=64**  **n (%)** | **Treatment-sensitive, n=53**  **n (%)** | **Relapsing, n=11**  **n (%)** | **p-value** |
| --- | --- | --- | --- | --- | --- |
|  | Sex  Male  Female | 45 (70)  19 (30) | 37 (70)  16 (30) | 8 (73)  3 (27) | NS |
|  | Age (years)  Median  Range | 54  28-65 | 54  28-65 | 58  40-64 | NS |
|  | DLBCL NOS  GCB  Non-GCB  Not determined  TCRB  Intravascular  FL grade 3B  Not reviewed | 53 (83)  26 (49)  21 (40)  6 (11)  2 (3)  1 (2)  3 (5)  5 (8) | 45 (85)  21 (47)  18 (40)  6 (13)  2 (4)  1 (2)  2 (4)  3 (6) | 8 (73)  5 (62)  3 (38)  0  0  0  1 (9)  2 (18) | NS  -  -  - |
|  | DHL  Yes  No  Not determined | 8 (12)  48 (75)  8 (12) | 7 (13)  40 (75)  6 (11) | 1 (9)  8 (73)  2 (18) | NS |
|  | ECOG  PS>1  PS≤1 | 26 (41)  38 (59) | 18 (34)  35 (66) | 8 (73)  3 (27) | 0.039 |
|  | Stage  II  III  IV | 5 (8)  16 (25)  43 (67) | 5 (9)  15 (28)  33 (62) | 0  1 (9)  10 (91) | NS |
|  | B-symptoms  Yes  No | 37 (58)  27 (42) | 29 (55)  24 (45) | 8 (73)  3 (27) | NS |
|  | LDH elevation  Yes  No | 57 (89)  7 (11) | 46 (87)  7 (13) | 11 (100)  0 | NS |
|  | aaIPI  0-1  2  3 | 5 (8)  37 (58)  22 (34) | 5 (9)  33 (62)  15 (28) | 0  4 (36)  7 (64) | 0.067 |
| **B)** | **Characteristics** | **All, n=31**  **n (%)** | **Treatment-sensitive, n=24**  **n (%)** | **Relapsing, n=7**  **n (%)** | **p-value** |
|  | Sex  Male  Female | 20 (65)  11 (35) | 17 (71)  7 (29) | 3 (43)  4 (57) | NS |
|  | Age (years)  Median  Range | 54  30-65 | 53  30-65 | 60  50-64 | NS |
|  | DLBCL NOS  GCB  Non-GCB  Not determined  TCRB  Intravascular  FL grade 3B  Not reviewed | 25 (81)  12 (48)  11 (44)  2 (0)  0  1 (3)  2 (6)  3 (10) | 11 (46)  8 (33)  2 (8)  0  1 (4)  1 (4)  1 (4) | 1 (14)  3 (43)  0  0  0  1 (14)  2 (29) | NS  -  - |
|  | DHL  Yes  No  Not determined | 3 (10)  23 (74)  5 (16) | 3 (12)  19 (79)  2 (8) | 0  4 (57)  3 (43) | NS |
|  | ECOG  PS$>$1  PS$\leq$1 | 10 (32)  21 (68) | 6 (25)  18 (75) | 4 (57)  3 (43) | NS |
|  | Stage  II  III  IV | 4 (13)  5 (16)  22 (71) | 4 (17)  4 (17)  16 (67) | 0  1 (14)  6 (86) | NS |
|  | B-symptoms  Yes  No | 18 (58)  13 (42) | 13 (54)  11 (46) | 5 (71)  2 (29) | NS |
|  | LDH increase  Yes  No | 29 (94)  2 (6) | 22 (92)  2 (8) | 7 (100)  0 | NS |
|  | aaIPI  0-1  2  3 | 3 (10)  18 (58)  10 (32) | 3 (12)  15 (62)  6 (25) | 0  3 (43)  4 (57) | NS |

Abbreviations: aaIPI, age-adjusted International Prognostic Index; DHL, double-hit lymphoma; DLBCL NOS, diffuse large B-cell lymphoma, not otherwise specified; ECOG, Eastern Cooperative Oncology Group; FL, follicular lymphoma; GCB, germinal center B-cell-like; LDH, lactate dehydrogenase; NS, not significant; PS, performance status; TCRB, T-cell rich B-cell lymphoma.

| **Table S2:** 190 proteins were differentially expressed in tumor tissue of relapsing and treatment-sensitive patients when using p<0.05 (n=64). | | | |
| --- | --- | --- | --- |
| **Fold change**  **(relapsing/**  **treatment-sensitive)** | **Protein name** | **Gene name** | **p-value** |
| **Upregulated** | | | |
| 2.40 | Peroxisomal membrane protein 11B | *PEX11B* | 0.002 |
| 2.35 | dCTP pyrophosphatase 1 | *DCTPP1* | 0.014 |
| 2.31 | SRA stem-loop-interacting RNA-binding protein, mitochondrial | *SLIRP* | 0.022 |
| 2.24 | Huntingtin-interacting protein K | *HYPK* | 0.002 |
| 2.23 | Nucleoplasmin-3 | *NPM3* | 0.037 |
| 2.17 | DNA replication licensing factor MCM6 | *MCM6* | 0.040 |
| 2.02 | DNA replication licensing factor MCM2 | *MCM2* | 0.049 |
| 1.97 | Succinate-semialdehyde dehydrogenase, mitochondrial | *ALDH5A1* | 0.032 |
| 1.95 | Exosome complex component MTR3 | *EXOSC6* | 0.001 |
| 1.87 | Spermidine synthase | *SRM* | 0.024 |
| 1.87 | 28S ribosomal protein S16, mitochondrial | *MRPS16* | 0.011 |
| 1.84 | Exosome complex component RRP40 | *EXOSC3* | 0.011 |
| 1.84 | Zinc finger protein 706 | *ZNF706* | 0.002 |
| 1.77 | Mitochondrial import inner membrane translocase subunit Tim13 | *TIMM13* | 0.014 |
| 1.75 | Cyclin-dependent kinase 2 | *CDK2* | 0.039 |
| 1.74 | DNA polymerase epsilon subunit 3 | *POLE3* | 0.018 |
| 1.73 | Serpin B8 | *SERPINB8* | 0.011 |
| 1.73 | NADH dehydrogenase [ubiquinone] 1 alpha subcomplex subunit 2 | *NDUFA2* | 0.015 |
| 1.72 | 39S ribosomal protein L17, mitochondrial | *MRPL17* | 0.004 |
| 1.72 | H/ACA ribonucleoprotein complex subunit 1 | *GAR1* | 0.022 |
| 1.71 | Serine--tRNA ligase, mitochondrial | *SARS2* | 0.002 |
| 1.69 | BAG family molecular chaperone regulator 2 | *BAG2* | 0.020 |
| 1.69 | Very-long-chain (3R)-3-hydroxyacyl-CoA dehydratase 3 | *HACD3* | 0.013 |
| 1.68 | 39S ribosomal protein L20, mitochondrial | *MRPL20* | 0.019 |
| 1.67 | U6 snRNA-associated Sm-like protein LSm4 | *LSM4* | 0.032 |
| 1.67 | 3-ketoacyl-CoA thiolase, mitochondrial | *ACAA2* | 0.004 |
| 1.66 | Quinone oxidoreductase | *CRYZ* | 0.040 |
| 1.66 | Prothymosin alpha; Prothymosin alpha; Thymosin alpha-1 | *PTMA* | 0.045 |
| 1.66 | 28S ribosomal protein S21, mitochondrial | *MRPS21* | 0.009 |
| 1.66 | 28S ribosomal protein S34, mitochondrial | *MRPS34* | 0.002 |
| 1.65 | Mitotic spindle-associated MMXD complex subunit MIP18 | *FAM96B* | 0.003 |
| 1.64 | MOB kinase activator 1A; 1B | *MOB1A;MOB1B* | 0.019 |
| 1.64 | Deoxycytidine kinase | *DCK* | 0.022 |
| 1.64 | 40S ribosomal protein S30 | *FAU* | 0.021 |
| 1.63 | NADH dehydrogenase [ubiquinone] 1 alpha subcomplex factor 3 | *NDUFAF3* | 0.033 |
| 1.63 | Protein Dr1 | *DR1* | 0.025 |
| 1.63 | Exosome complex component RRP4 | *EXOSC2* | 0.023 |
| 1.62 | 60S ribosomal protein L19 | *RPL19* | 0.031 |
| 1.62 | TP53-regulating kinase | *TP53RK* | 0.014 |
| 1.61 | 39S ribosomal protein L18, mitochondrial | *MRPL18* | 0.028 |
| 1.60 | Mitochondrial import receptor subunit TOM22 homolog | *TOMM22* | 0.021 |
| 1.60 | Putative transferase CAF17, mitochondrial | *IBA57* | 0.002 |
| 1.60 | Enhancer of rudimentary homolog | *ERH* | 0.008 |
| 1.59 | Rho GTPase-activating protein 17 | *ARHGAP17* | 0.008 |
| 1.59 | Serine/threonine-protein kinase 4 | *STK4* | 0.030 |
| 1.59 | Cytosolic Fe-S cluster assembly factor NUBP2 | *NUBP2* | 0.006 |
| 1.59 | Acidic leucine-rich nuclear phosphoprotein 32 family member A | *ANP32A* | 0.011 |
| 1.59 | 39S ribosomal protein L14, mitochondrial | *MRPL14* | 0.005 |
| 1.58 | CDKN2A-interacting protein | *CDKN2AIP* | 0.003 |
| 1.58 | Enoyl-CoA delta isomerase 1, mitochondrial | *ECI1* | 0.018 |
| 1.56 | 28S ribosomal protein S23, mitochondrial | *MRPS23* | 0.009 |
| 1.55 | 39S ribosomal protein L53, mitochondrial | *MRPL53* | 0.015 |
| 1.55 | Secretory carrier-associated membrane protein 2 | *SCAMP2* | 0.043 |
| 1.55 | 60S ribosomal protein L35a | *RPL35A* | 0.026 |
| 1.55 | Stromal cell-derived factor 2-like protein 1 | *SDF2L1* | 0.018 |
| 1.54 | Exosome complex component RRP42 | *EXOSC7* | 0.025 |
| 1.54 | 39S ribosomal protein L19, mitochondrial | *MRPL19* | 0.033 |
| 1.53 | Exosome complex component CSL4 | *EXOSC1* | 0.022 |
| 1.53 | Voltage-dependent anion-selective channel protein 3 | *VDAC3* | 0.021 |
| 1.53 | Nucleoporin Nup43 | *NUP43* | 0.025 |
| 1.51 | Medium-chain specific acyl-CoA dehydrogenase, mitochondrial | *ACADM* | 0.047 |
| 1.51 | SUMO-conjugating enzyme UBC9 | *UBE2I* | 0.045 |
| 1.51 | Coiled-coil-helix-coiled-coil-helix domain-containing protein 1 | *CHCHD1* | 0.045 |
| 1.51 | SUMO-activating enzyme subunit 2 | *UBA2* | 0.002 |
| 1.51 | Peptidyl-prolyl cis-trans isomerase FKBP3 | *FKBP3* | 0.044 |
| 1.51 | Mannose-P-dolichol utilization defect 1 protein | *MPDU1* | 0.031 |
| 1.50 | 28S ribosomal protein S28, mitochondrial | *MRPS28* | 0.034 |
| 1.50 | Derlin-1 | *DERL1* | 0.020 |
| 1.50 | Polymerase delta-interacting protein 2 | *POLDIP2* | 0.029 |
| 1.50 | Histone H2A.V; Histone H2A.Z | *H2AFV;H2AFZ* | 0.028 |
| 1.50 | Phosphoglycolate phosphatase | *PGP* | 0.037 |
| 1.50 | Glia maturation factor gamma | *GMFG* | 0.031 |
| 1.48 | Stathmin | *STMN1* | 0.043 |
| 1.48 | 40S ribosomal protein S20 | *RPS20* | 0.023 |
| 1.48 | Eukaryotic translation elongation factor 1 epsilon-1 | *EEF1E1* | 0.021 |
| 1.47 | 60S ribosomal protein L27a | *RPL27A* | 0.004 |
| 1.47 | Scaffold attachment factor B1 | *SAFB* | 0.014 |
| 1.46 | 60S ribosomal protein L36 | *RPL36* | 0.029 |
| 1.46 | Cytochrome c oxidase assembly factor 6 homolog | *COA6* | 0.041 |
| 1.46 | Aflatoxin B1 aldehyde reductase member 2 | *AKR7A2* | 0.025 |
| 1.46 | 60S ribosomal protein L18 | *RPL18* | 0.007 |
| 1.46 | Pro-interleukin-16;Interleukin-16 | *IL16* | 0.040 |
| 1.45 | Growth arrest and DNA damage-inducible interacting protein 1 | *GADD45GIP1* | 0.018 |
| 1.45 | 40S ribosomal protein S14 | *RPS14* | 0.020 |
| 1.45 | Bcl-2-like protein 13 | *BCL2L13* | 0.040 |
| 1.45 | Ran-specific GTPase-activating protein | *RANBP1* | 0.022 |
| 1.44 | 40S ribosomal protein S25 | *RPS25* | 0.017 |
| 1.44 | 39S ribosomal protein L49, mitochondrial | *MRPL49* | 0.041 |
| 1.44 | U6 snRNA-associated Sm-like protein LSm2 | *LSM2* | 0.043 |
| 1.44 | 39S ribosomal protein L12, mitochondrial | *MRPL12* | 0.049 |
| 1.44 | Electron transfer flavoprotein subunit beta | *ETFB* | 0.011 |
| 1.43 | Chromatin accessibility complex protein 1 | *CHRAC1* | 0.043 |
| 1.43 | Annexin A11 | *ANXA11* | 0.024 |
| 1.43 | Peptidyl-prolyl cis-trans isomerase-like 1 | *PPIL1* | 0.036 |
| 1.42 | 40S ribosomal protein SA | *RPSA* | 0.017 |
| 1.41 | 60S ribosomal protein L22 | *RPL22* | 0.009 |
| 1.41 | Nucleoporin SEH1 | *SEH1L* | 0.025 |
| 1.41 | 40S ribosomal protein S17 | *RPS17* | 0.019 |
| 1.41 | Acidic leucine-rich nuclear phosphoprotein 32 family member E | *ANP32E* | 0.029 |
| 1.41 | Golgi to ER traffic protein 4 homolog | *GET4* | 0.045 |
| 1.41 | RNA-binding protein 8A | *RBM8A* | 0.032 |
| 1.40 | 5-formyltetrahydrofolate cyclo-ligase | *MTHFS* | 0.048 |
| 1.40 | 60S ribosomal protein L38 | *RPL38* | 0.021 |
| 1.40 | Ras-related C3 botulinum toxin substrate 1; substrate 3 | *RAC1;RAC3* | 0.007 |
| 1.40 | Protein phosphatase 1 regulatory subunit 11 | *PPP1R11* | 0.048 |
| 1.40 | 40S ribosomal protein S9 | *RPS9* | 0.009 |
| 1.40 | 60S ribosomal protein L13a | *RPL13A* | 0.029 |
| 1.40 | 60S ribosomal protein L11 | *RPL11* | 0.008 |
| 1.39 | Platelet-activating factor acetylhydrolase IB subunit gamma | *PAFAH1B3* | 0.020 |
| 1.38 | SAFB-like transcription modulator | *SLTM* | 0.027 |
| 1.38 | Protein SET | *SET* | 0.033 |
| 1.38 | 40S ribosomal protein S12 | *RPS12* | 0.007 |
| 1.38 | 10 kDa heat shock protein, mitochondrial | *HSPE1* | 0.043 |
| 1.37 | 60S ribosomal protein L27 | *RPL27* | 0.026 |
| 1.37 | ER membrane protein complex subunit 2 | *EMC2* | 0.021 |
| 1.37 | Chromatin target of PRMT1 protein | *CHTOP* | 0.043 |
| 1.37 | Peptidyl-tRNA hydrolase 2, mitochondrial | *PTRH2* | 0.047 |
| 1.36 | Hematological and neurological expressed 1-like protein | *HN1L* | 0.019 |
| 1.36 | NEDD8-conjugating enzyme Ubc12 | *UBE2M* | 0.038 |
| 1.36 | Small nuclear ribonucleoprotein Sm D3 | *SNRPD3* | 0.023 |
| 1.36 | 60S ribosomal protein L18a | *RPL18A* | 0.039 |
| 1.36 | 40S ribosomal protein S16 | *RPS16* | 0.029 |
| 1.36 | ADP-ribosylation factor-like protein 2 | *ARL2* | 0.043 |
| 1.36 | 60S ribosomal protein L12 | *RPL12* | 0.019 |
| 1.36 | Lactoylglutathione lyase | *GLO1* | 0.045 |
| 1.36 | 40S ribosomal protein S13 | *RPS13* | 0.037 |
| 1.35 | GH3 domain-containing protein | *GHDC* | 0.034 |
| 1.35 | Serine/threonine-protein phosphatase PP1-beta catalytic subunit | *PPP1CB* | 0.039 |
| 1.35 | ADP-ribosylation factor 6 | *ARF6* | 0.039 |
| 1.35 | GTP-binding nuclear protein Ran | *RAN* | 0.009 |
| 1.35 | Charged multivesicular body protein 4a | *CHMP4A* | 0.040 |
| 1.34 | 40S ribosomal protein S18 | *RPS18* | 0.026 |
| 1.34 | Serine/arginine-rich splicing factor 11 | *SRSF11* | 0.021 |
| 1.33 | 40S ribosomal protein S3 | *RPS3* | 0.002 |
| 1.32 | NSFL1 cofactor p47 | *NSFL1C* | 0.035 |
| 1.32 | Y-box-binding protein 3 | *YBX3* | 0.022 |
| 1.32 | Dolichyl-diphosphooligosaccharide glycosyltransferase DAD1 | *DAD1* | 0.045 |
| 1.31 | 40S ribosomal protein S4, X isoform | *RPS4X* | 0.026 |
| 1.31 | 40S ribosomal protein S8 | *RPS8* | 0.039 |
| 1.30 | Thioredoxin-dependent peroxide reductase, mitochondrial | *PRDX3* | 0.045 |
| 1.29 | 60S acidic ribosomal protein P0; 60S acidic ribosomal P0-like | *RPLP0;RPLP0P6* | 0.025 |
| 1.28 | Vesicle-associated membrane protein-associated protein B/C | *VAPB* | 0.040 |
| 1.28 | Rab-like protein 6 | *RABL6* | 0.033 |
| 1.27 | Small nuclear ribonucleoprotein Sm D2 | *SNRPD2* | 0.042 |
| 1.27 | Eukaryotic translation initiation factor 3 subunit K | *EIF3K* | 0.007 |
| 1.26 | Crk-like protein | *CRKL* | 0.032 |
| 1.24 | Membrane-associated progesterone receptor component 2 | *PGRMC2* | 0.023 |
| 1.22 | Interleukin enhancer-binding factor 2 | *ILF2* | 0.017 |
| 1.22 | Eukaryotic translation initiation factor 2 subunit 1 | *EIF2S1* | 0.021 |
| **Downregulated** | | | |
| 0.28 | Protein Niban | *FAM129A* | 0.002 |
| 0.28 | Tryptase alpha/beta-1; Tryptase beta-2 | *TPSAB1;TPSB2* | 0.042 |
| 0.30 | Hexokinase-3 | *HK3* | 0.028 |
| 0.32 | 26S proteasome non-ATPase regulatory subunit 2 | *PSMD2* | <0.001 |
| 0.32 | Arachidonate 5-lipoxygenase | *ALOX5* | 0.001 |
| 0.33 | L-amino-acid oxidase | *IL4I1* | 0.006 |
| 0.34 | Dihydropyrimidinase-related protein 3 | *DPYSL3* | 0.001 |
| 0.36 | Branched-chain-amino-acid aminotransferase, cytosolic | *BCAT1* | 0.033 |
| 0.37 | Fructose-1,6-bisphosphatase 1 | *FBP1* | 0.041 |
| 0.38 | Pigment epithelium-derived factor | *SERPINF1* | 0.036 |
| 0.39 | Ribosome-binding protein 1 | *RRBP1* | 0.050 |
| 0.39 | Plectin | *PLEC* | 0.045 |
| 0.42 | Splicing factor 3B subunit 1 | *SF3B1* | 0.030 |
| 0.42 | Fibronectin; Anastellin; Ugl-Y1; Ugl-Y2; Ugl-Y3 | *FN1* | 0.044 |
| 0.42 | Proteasome subunit beta type-5 | *PSMB5* | 0.002 |
| 0.43 | Fibulin-2 | *FBLN2* | 0.042 |
| 0.43 | Plastin-3 | *PLS3* | 0.012 |
| 0.44 | Antithrombin-III | *SERPINC1* | 0.048 |
| 0.45 | Alpha-2-HS-glycoprotein; chain A; chain B | *AHSG* | 0.048 |
| 0.51 | DnaJ homolog subfamily A member 1 | *DNAJA1* | 0.012 |
| 0.51 | Retinoid-inducible serine carboxypeptidase | *SCPEP1* | 0.016 |
| 0.52 | Cell cycle and apoptosis regulator protein 2 | *CCAR2* | 0.007 |
| 0.52 | Annexin A1 | *ANXA1* | 0.003 |
| 0.55 | Ig gamma-3 chain C region | *IGHG3* | 0.019 |
| 0.56 | EH domain-containing protein 4 | *EHD4* | 0.004 |
| 0.58 | Rab GTPase-activating protein 1-like | *RABGAP1L* | 0.027 |
| 0.61 | Glutamine synthetase | *GLUL* | 0.047 |
| 0.61 | Eukaryotic translation initiation factor 3 subunit J | *EIF3J* | 0.038 |
| 0.63 | Myosin-9 | *MYH9* | 0.030 |
| 0.63 | Constitutive coactivator of PPAR-gamma-like protein 1 | *FAM120A* | 0.014 |
| 0.65 | V-type proton ATPase subunit E 1 | *ATP6V1E1* | 0.025 |
| 0.65 | Beta-hexosaminidase subunit beta chain B | *HEXB* | 0.006 |
| 0.65 | Tyrosine-tRNA ligase, cytoplasmic; cytoplasmic | *YARS* | 0.042 |
| 0.66 | Endoplasmic reticulum resident protein 44 | *ERP44* | 0.016 |
| 0.66 | V-type proton ATPase subunit H | *ATP6V1H* | 0.043 |
| 0.68 | Filamin-A | *FLNA* | 0.042 |
| 0.69 | Syntaxin-binding protein 2 | *STXBP2* | 0.046 |
| 0.73 | Moesin | *MSN* | 0.024 |
| 0.75 | Glyceraldehyde-3-phosphate dehydrogenase | *GAPDH* | 0.012 |
| 0.75 | Transketolase | *TKT* | 0.007 |
| 0.79 | L-lactate dehydrogenase A chain | *LDHA* | 0.012 |

| **Table S3:** 64 proteins were differentially expressed in tumor tissue from relapsing and treatment-sensitive patients from the high-risk cluster when using p<0.05 (n=19). | | | |
| --- | --- | --- | --- |
| **Fold change (relapsing/**  **treatment-sensitive)** | **Protein name** | **Gene name** | **p-value** |
| **Upregulated** |  |  |  |
| 3.84 | Aldehyde dehydrogenase, mitochondrial | *ALDH2* | 0.011 |
| 3.11 | Hypoxia up-regulated protein 1 | *HYOU1* | 0.022 |
| 2.96 | CD82 antigen | *CD82* | 0.036 |
| 2.47 | Methylmalonate-semialdehyde dehydrogenase [acylating] | *ALDH6A1* | 0.040 |
| 2.38 | Mitochondrial thiamine pyrophosphate carrier | *SLC25A19* | 0.017 |
| 2.37 | Aldehyde dehydrogenase family 16 member A1 | *ALDH16A1* | 0.026 |
| 2.35 | Golgi phosphoprotein 3 | *GOLPH3* | 0.043 |
| 2.13 | Aminopeptidase B | *RNPEP* | 0.010 |
| 2.11 | MICOS complex subunit MIC19 | *CHCHD3* | 0.016 |
| 1.99 | CDP-diacylglycerol--inositol 3-phosphatidyltransferase | *CDIPT* | 0.026 |
| 1.95 | Ras-related protein Rab-35 | *RAB35* | 0.031 |
| 1.88 | Tapasin | *TAPBP* | 0.039 |
| 1.80 | Annexin A4 | *ANXA4* | 0.003 |
| 1.79 | Stromal cell-derived factor 2-like protein 1 | *SDF2L1* | 0.019 |
| 1.77 | Sorting nexin-6;Sorting nexin-6, N-terminally processed | *SNX6* | 0.013 |
| 1.77 | Epoxide hydrolase 1 | *EPHX1* | 0.043 |
| 1.74 | Alpha-soluble NSF attachment protein | *NAPA* | 0.032 |
| 1.71 | Voltage-dependent anion-selective channel protein 3 | *VDAC3* | 0.013 |
| 1.70 | Very long-chain specific acyl-CoA dehydrogenase | *ACADVL* | 0.032 |
| 1.69 | Putative transferase CAF17, mitochondrial | *IBA57* | 0.002 |
| 1.69 | Estradiol 17-beta-dehydrogenase 11 | *HSD17B11* | 0.025 |
| 1.68 | Microsomal glutathione S-transferase 3 | *MGST3* | 0.044 |
| 1.64 | Nicalin | *NCLN* | 0.027 |
| 1.60 | Aminoacyl tRNA synthase complex-interacting protein 1 | *AIMP1* | 0.049 |
| 1.59 | Phosphatidate cytidylyltransferase 2 | *CDS2* | 0.030 |
| 1.58 | NADH-cytochrome b5 reductase 1 | *CYB5R1* | 0.034 |
| 1.58 | Probable ATP-dependent RNA helicase DDX6 | *DDX6* | 0.045 |
| 1.57 | 28S ribosomal protein S22, mitochondrial | *MRPS22* | 0.043 |
| 1.51 | 28S ribosomal protein S21, mitochondrial | *MRPS21* | 0.046 |
| 1.48 | LEM domain-containing protein 2 | *LEMD2* | 0.016 |
| 1.46 | Atlastin-3 | *ATL3* | 0.012 |
| 1.46 | Platelet-activating factor acetylhydrolase IB subunit alpha | *PAFAH1B1* | 0.020 |
| 1.44 | CDKN2A-interacting protein | *CDKN2AIP* | 0.043 |
| 1.44 | Rho-related GTP-binding protein RhoG | *RHOG* | 0.018 |
| 1.44 | 40S ribosomal protein S11 | *RPS11* | 0.039 |
| 1.43 | Lysosomal Pro-X carboxypeptidase | *PRCP* | 0.047 |
| 1.40 | Cilia- and flagella-associated protein 20 | *CFAP20* | 0.030 |
| 1.38 | Vesicular integral-membrane protein VIP36 | *LMAN2* | 0.027 |
| 1.38 | Ras-related C3 botulinum toxin substrate 1;3 | *RAC1;RAC3* | 0.021 |
| 1.36 | Nucleobindin-1 | *NUCB1* | 0.043 |
| 1.30 | Vesicle-associated membrane protein-associated protein B/C | *VAPB* | 0.021 |
| 1.25 | 3-hydroxyisobutyrate dehydrogenase, mitochondrial | *HIBADH* | 0.025 |
| 1.25 | Electron transfer flavoprotein subunit alpha, mitochondrial | *ETFA* | 0.038 |
| **Downregulated** | | | |
| 0.37 | Proteasome subunit beta type-5 | *PSMB5* | 0.019 |
| 0.38 | 40S ribosomal protein S4, Y isoform 1 | *RPS4Y1* | 0.036 |
| 0.43 | 60S ribosomal protein L8 | *RPL8* | 0.003 |
| 0.43 | Insulin-like growth factor 2 mRNA-binding protein 3 | *IGF2BP3* | 0.045 |
| 0.43 | RNA-binding protein 3 | *RBM3* | 0.035 |
| 0.44 | Caspase-7;Caspase-7 subunit p20;Caspase-7 subunit p11 | *CASP7* | <0.001 |
| 0.45 | Proteasome subunit beta type-6 | *PSMB6* | 0.020 |
| 0.50 | Integrin-linked kinase-associated ser/thr phosphatase 2C | *ILKAP* | 0.012 |
| 0.53 | U6 snRNA-associated Sm-like protein LSm8 | *LSM8* | 0.032 |
| 0.55 | Zinc finger protein 428 | *ZNF428* | 0.039 |
| 0.57 | Ezrin | *EZR* | 0.044 |
| 0.60 | 26S proteasome non-ATPase regulatory subunit 2 | *PSMD2* | 0.047 |
| 0.60 | tRNA (guanine-N(7)-)-methyltransferase non-catalytic WDR4 | *WDR4* | 0.048 |
| 0.64 | Proteasome subunit beta type-7 | *PSMB7* | 0.019 |
| 0.66 | Lysosome-associated membrane glycoprotein 1 | *LAMP1* | 0.018 |
| 0.67 | 60S ribosomal protein L10 | *RPL10* | 0.047 |
| 0.68 | WAS/WASL-interacting protein family member 1 | *WIPF1* | 0.022 |
| 0.72 | Serine/arginine-rich splicing factor 9 | *SRSF9* | 0.038 |
| 0.72 | Methylosome protein 50 | *WDR77* | 0.043 |
| 0.78 | T-complex protein 1 subunit beta | *CCT2* | 0.030 |
| 0.79 | Small nuclear ribonucleoprotein-associated proteins B;N | *SNRPB;SNRPN* | 0.036 |

**Table S4: IPA Canonical Pathways for tumor tissue**

| **Ingenuity Canonical Pathways** | **p-value** | **Ratio** | **z-score** | **Gene Name** |
| --- | --- | --- | --- | --- |
| EIF2 Signaling | <0.001 | 0.265 | 3.742 | *EIF2S1, EIF3J, EIF3K, FAU, PPP1CB, RPL11, RPL12, RPL13A, RPL18, RPL18A, RPL19, RPL22, RPL27, RPL27A, RPL35A, RPL36, RPL38, RPLP0, RPS12, RPS13, RPS14, RPS16, RPS17, RPS18, RPS20, RPS25, RPS3, RPS4X, RPS8, RPS9, RPSA* |
| Coronavirus Pathogenesis Pathway | <0.001 | 0.254 | -3.873 | *CDK2, FAU, RPS12, RPS13, RPS14, RPS16, RPS17, RPS18, RPS20, RPS25, RPS3, RPS4X, RPS8, RPS9, RPSA* |
| mTOR Signaling | <0.001 | 0.233 |  | *EIF3J, EIF3K, FAU, RAC1, RPS12, RPS13, RPS14, RPS16, RPS17, RPS18, RPS20, RPS25, RPS3, RPS4X, RPS8, RPS9, RPSA* |
| Regulation of eIF4 and p70S6K Signaling | <0.001 | 0.224 |  | *EIF2S1, EIF3J, EIF3K, FAU, RPS12, RPS13, RPS14, RPS16, RPS17, RPS18, RPS20, RPS25, RPS3, RPS4X, RPS8, RPS9, RPSA* |
| Leucine Degradation I | 0.025 | 0.667 |  | *ACADM, BCAT1* |
| Role of p14/p19ARF in Tumor Suppression | 0.047 | 0.500 |  | *NPM3, RAC1* |

2,026 proteins identified in at least 70% of tumor samples in each response group along with expression data were submitted to IPA. The ratio is the proportion of molecules in the data from the canonical pathway relative to all the molecules in that pathway. Z-scores $\geq$ |2| are considered statistically significant. A positive z-score indicates that the pathway is predicted to be activated, while a negative z-score indicates that the pathway is predicted to be inhibited. Missing z-scores indicate a pathway for which the z-score cannot be calculated. For simplicity, proteins are referred to by gene names.

**Table S5: IPA Disease and Function Analysis for tumor tissue.** 2,026 proteins identified in at least 70% of samples in each response group in all 64 tumor tissue samples along with expression data were submitted to IPA. See Excel document for Table S5.

**Table S6: IPA Network Analysis for tumor tissue.** 2,026 proteins identified in at least 70% of samples in each response group in all 64 tumor tissue samples along with expression data were submitted to IPA. See Excel document for Table S6.

**Table S7:** **IPA Canonical Pathways for tumor tissue from the high-risk cluster.**

| **Ingenuity Canonical Pathways** | **p-value** | **Ratio** | **z-score** | **Gene Name** |
| --- | --- | --- | --- | --- |
| Xenobiotic Metabolism AHR Signaling Pathway | 0.002 | 0.222E | 2.000 | *ALDH16A1, ALDH2, ALDH6A1, MGST3* |
| LPS/IL-1 Mediated Inhibition of RXR Function | 0.004 | 0.174 |  | *ALDH16A1, ALDH2, ALDH6A1, MGST3* |
| Xenobiotic Metabolism CAR Signaling Pathway | 0.008 | 0.148 | 2.000 | *ALDH16A1, ALDH2, ALDH6A1, MGST3* |
| Aryl Hydrocarbon Receptor Signaling | 0.009 | 0.143 |  | *ALDH16A1, ALDH2, ALDH6A1, MGST3* |
| Sphingosine-1-phosphate Signaling | 0.015 | 0.167 |  | *CASP7, RAC1, RHOG* |
| Xenobiotic Metabolism PXR Signaling Pathway | 0.018 | 0.118 | 2.000 | *ALDH16A1, ALDH2, ALDH6A1, MGST3* |
| Valine Degradation I | 0.028 | 0.222 |  | *ALDH6A1, HIBADH* |
| β-alanine Degradation I | 0.030 | 1 |  | *ALDH6A1* |
| Phenylethylamine Degradation I | 0.030 | 1 |  | *ALDH2* |
| FAT10 Signaling Pathway | 0.031 | 0.100 |  | *PSMB5, PSMB6, PSMB7, PSMD2* |
| Antigen Presentation Pathway | 0.034 | 0.125 |  | *PSMB5, PSMB6, TAPBP* |
| Xenobiotic Metabolism Signaling | 0.045 | 0.089 |  | *ALDH16A1, ALDH2, ALDH6A1, MGST3* |
| Inhibition of ARE-Mediated mRNA Degradation Pathway | 0.050 | 0.075 |  | *DDX6, PSMB5, PSMB6, PSMB7, PSMD2* |

2,123 proteins identified in at least 70% of tumor samples in each response group from samples in the high-risk cluster along with expression data were submitted to IPA. The ratio is the proportion of molecules in the data from the canonical pathway relative to all the molecules in that pathway. Z-scores $\geq$ |2| are considered statistically significant. A positive z-score indicates that the pathway is predicted to be activated, while a negative z-score indicates that the pathway is predicted to be inhibited. Missing z-scores indicate a pathway for which the z-score cannot be calculated. For simplicity, proteins are referred to by gene names.

**Table S8: IPA Disease and Function Analysis for high-risk cluster identified in tumor tissue.** 2,123 proteins identified in at least 70% of samples in each response group in 19 tumor tissue samples (10 relapsing, 9 treatment-sensitive) from the high-risk cluster along with expression data were submitted to IPA. See Excel document for Table S8.

**Table S9: IPA Network Analysis for high-risk cluster identified in tumor tissue.** 2,123 proteins identified in at least 70% of samples in each response group in 19 tumor tissue samples (10 relapsing, 9 treatment-sensitive) from the high-risk cluster along with expression data were submitted to IPA. See Excel document for Table S9.

| **Table S10:** 20 proteins were differentially expressed in serum samples from relapsing and treatment-sensitive patients when using p<0.05. | | | |
| --- | --- | --- | --- |
| **Fold change (relapsing/**  **treatment-sensitive)** | **Protein name** | **Gene name** | **p-value** |
| **Upregulated** | | | |
| 5.72 | Serum amyloid A-1 protein | *SAA1* | 0.034 |
| 4.67 | Fibrinogen-like protein 1 | *FGL1* | 0.035 |
| 3.31 | Apolipoprotein C-II; Proapolipoprotein C-II | *APOC2* | 0.011 |
| 3.04 | Coagulation factor VIII; Factor VIIIa heavy chain | *F8* | 0.004 |
| 2.76 | Hepatocyte growth factor-like; macrophage stimulating protein | *MST1;MST1L* | 0.007 |
| 2.58 | Fibrillin-1 | *FBN1* | 0.026 |
| 2.39 | Ig kappa chain V-III region B6 | *IGKV3-20* | 0.022 |
| 2.35 | Cholesteryl ester transfer protein | *CETP* | 0.013 |
| 2.20 | Complement factor H-related protein 3 | *CFHR3* | 0.019 |
| 2.12 | Out at first protein homolog | *OAF* | 0.013 |
| 1.78 | Ficolin-3 | *FCN3* | 0.014 |
| 1.71 | Phospholipid transfer protein | *PLTP* | 0.043 |
| 1.64 | Inter-alpha-trypsin inhibitor heavy chain H3 | *ITIH3* | 0.007 |
| 1.61 | Dickkopf-related protein 3 | *DKK3* | 0.032 |
| 1.60 | ADAMTS-like protein 4 | *ADAMTSL4* | 0.036 |
| **Downregulated** | | | |
| 0.18 | Lactotransferrin | *LTF* | 0.005 |
| 0.26 | Fatty acid-binding protein, epidermal | *FABP5* | 0.043 |
| 0.51 | Plasminogen activator inhibitor 1 | *SERPINE1* | 0.047 |
| 0.52 | DnaJ homolog subfamily B member 11 | *DNAJB11* | 0.023 |
| 0.57 | Angiogenin | *ANG* | 0.018 |

**Table S11:** **IPA Canonical Pathways for serum samples**

| **Ingenuity Canonical Pathways** | **p-value** | **Ratio** | **Gene Name** |
| --- | --- | --- | --- |
| LPS/IL-1 Mediated Inhibition of RXR Function | 0.003 | 0.4 | *APOC2, CETP, FABP5, PLTP* |
| Senescence Pathway | 0.040 | 0.4 | *SAA1, SERPINE1* |
|  |  |  |  |

298 proteins identified in at least 70% of serum samples in each response group along with expression data were submitted to IPA. The ratio is the proportion of molecules in the data from the canonical pathway relative to all the molecules in that pathway. Z-scores $\geq$ |2| are considered statistically significant. A positive z-score indicates that the pathway is predicted to be activated, while a negative z-score indicates that the pathway is predicted to be inhibited. Missing z-scores indicate a pathway for which the z-score cannot be calculated. For simplicity, proteins are referred to by gene names.

**Table S12: IPA Network Analysis for serum samples**. 298 proteins identified in at least 70% of samples in each response group in all 31 serum samples along with expression data were submitted to IPA. See Excel document for Table S12.
