# Supplementary figures and images for: Proteomics uncovers molecular features for relapse risk stratification in patients with diffuse large B-cell lymphoma
Source: Blood Cancer J. 2023 Oct 26;13(1):161. doi: 10.1038/s41408-023-00931-6 (PMC10603067; doi:10.1038/s41408-023-00931-6)

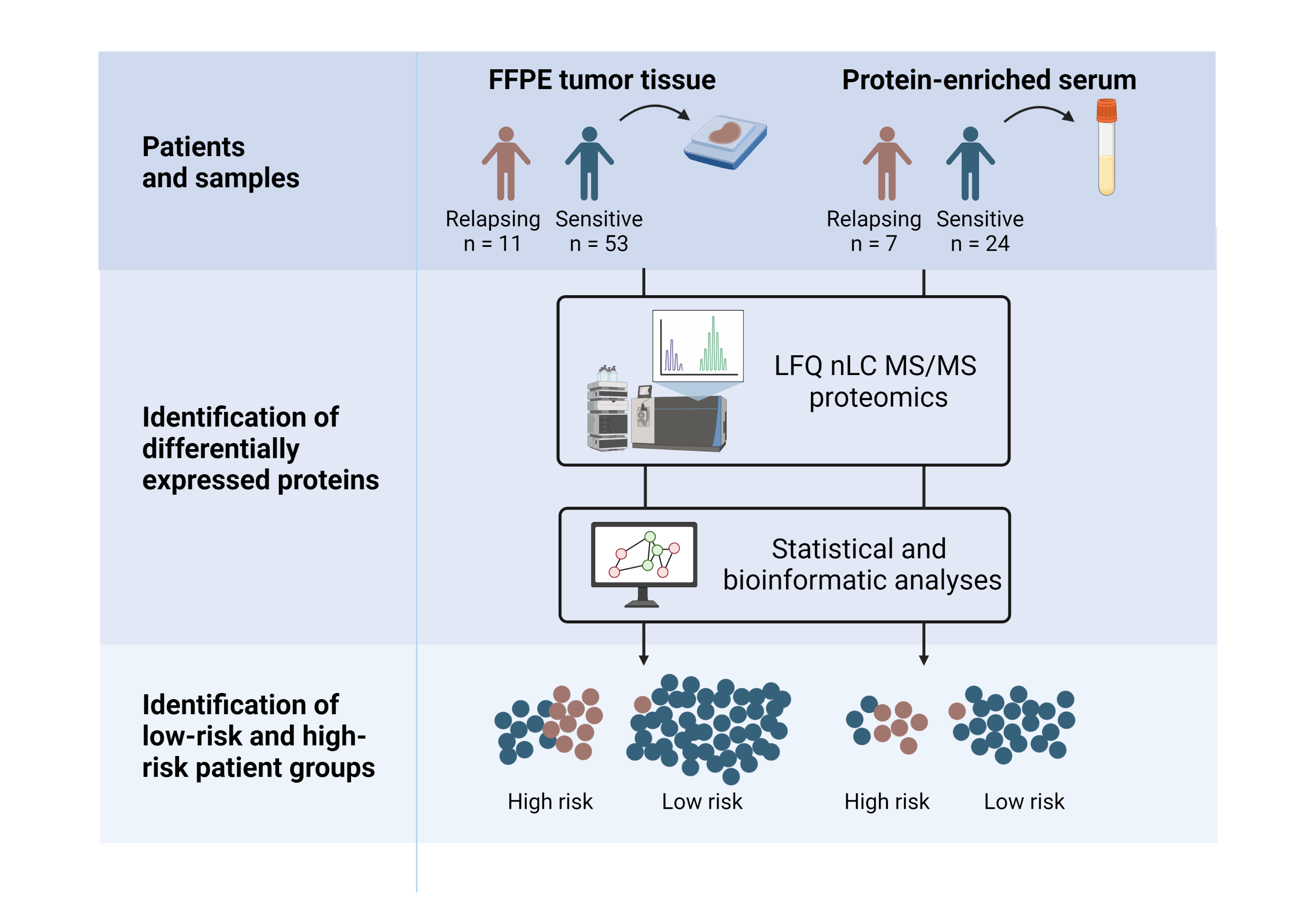

Supplement: Supplementary file 2 — Supplementary Figure 1 [file 41408_2023_931_MOESM2_ESM.png]

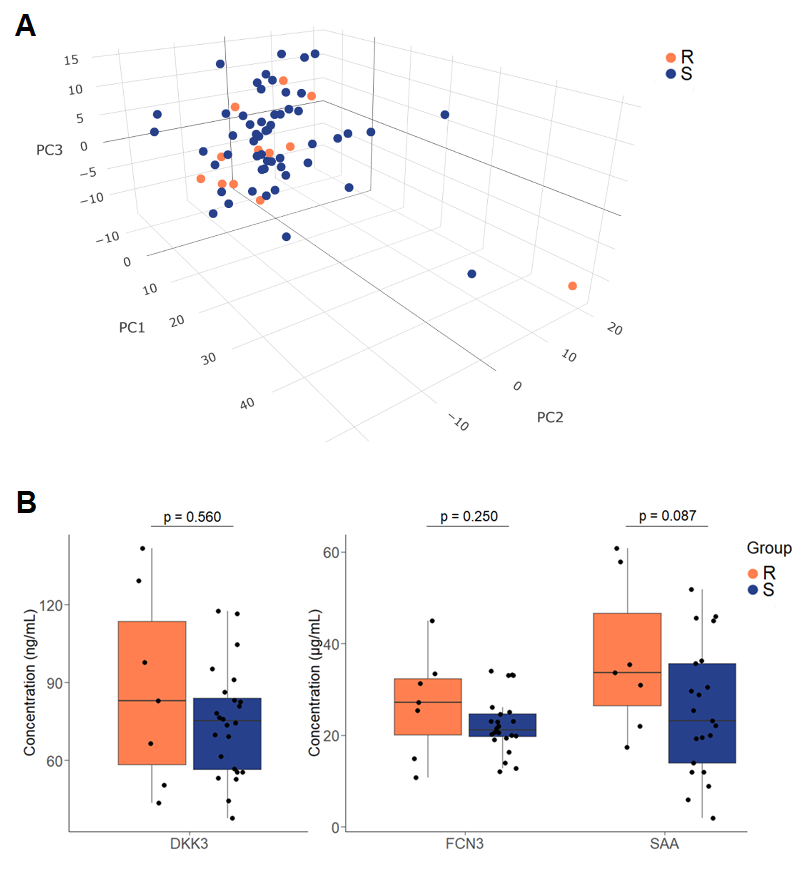

Supplement: Supplementary file 3 — Supplementary Figure 2 [file 41408_2023_931_MOESM3_ESM.png]
